# Structural and Functional Analysis of BipA, a Regulator of Virulence in Enteropathogenic Escherichia coli*

**DOI:** 10.1074/jbc.M115.659136

**Published:** 2015-07-10

**Authors:** Haitian Fan, Joseph Hahm, Stephen Diggs, J. Jefferson P. Perry, Gregor Blaha

**Affiliations:** From the Department of Biochemistry, University of California, Riverside, California 92521

**Keywords:** small GTPase, structural biology, structure-function, thermodynamics, translation regulation, translational regulation

## Abstract

The translational GTPase BipA regulates the expression of virulence and pathogenicity factors in several eubacteria. BipA-dependent expression of virulence factors occurs under starvation conditions, such as encountered during infection of a host. Under these conditions, BipA associates with the small ribosomal subunit. BipA also has a second function to promote the efficiency of late steps in biogenesis of large ribosomal subunits at low temperatures, presumably while bound to the ribosome. During starvation, the cellular concentration of stress alarmone guanosine-3′, 5′-bis pyrophosphate (ppGpp) is increased. This increase allows ppGpp to bind to BipA and switch its binding specificity from ribosomes to small ribosomal subunits. A conformational change of BipA upon ppGpp binding could explain the ppGpp regulation of the binding specificity of BipA. Here, we present the structures of the full-length BipA from *Escherichia coli* in apo, GDP-, and ppGpp-bound forms. The crystal structure and small-angle x-ray scattering data of the protein with bound nucleotides, together with a thermodynamic analysis of the binding of GDP and of ppGpp to BipA, indicate that the ppGpp-bound form of BipA adopts the structure of the GDP form. This suggests furthermore, that the switch in binding preference only occurs when both ppGpp and the small ribosomal subunit are present. This molecular mechanism would allow BipA to interact with both the ribosome and the small ribosomal subunit during stress response.

## Teaser

**Background:** BipA binds to ribosomes during exponential growth, but to small ribosomal subunits during starvation.

**Results:** We present the structure and thermodynamic analysis of GDP and stress alarmone guanosine-3′, 5′-bis pyrophosphate (ppGpp) binding to BipA.

**Conclusion:** Structures of GDP- and ppGpp-bound BipA are equivalent.

**Significance:** BipA switches its binding specificity only in the presence of both small ribosomal subunits and ppGpp.

## Introduction

BipA (TypA or YihK) is a member of the translational GTPase protein family, to which translational factors EF-G,^2^ EF-Tu, and IF2 also belong (1, 2). It is present only in eubacteria with a genome size larger than 2.8 × 10^6^ bp (2). Among these eubacteria are many plant and insect symbionts, as well as many plant, animal, and human pathogens (3).

In enteropathogenic *Escherichia coli*, BipA is essential for the regulation of multiple cell surface and virulence-associated components (4–6). Regulation of many virulence factors, including those of enteropathogenic *E. coli* (7), is integrated into a global cellular response that utilizes the alarmone ppGpp (8). The cellular concentration of ppGpp increases dramatically in response to starvation (9), which reshapes the transcriptome, stalls replication, and modulates translation (10). Upon starvation, BipA binds to the small ribosomal subunit (11), pointing to an allosteric regulation of BipA by ppGpp (11). Moreover, BipA is critical for efficient biogenesis of large ribosomal subunits at low temperatures (12), and it displays in its GTP-bound form a binding preference for ribosomes (11, 13). As these functions involve interactions with either the small or the large ribosomal subunit, they are mutually exclusive. To discern the molecular mechanism of regulation of BipA, we determined the effect of ppGpp binding on the molecular structure of BipA by x-ray crystallography, isothermal titration calorimetry (ITC) and small-angle x-ray scattering (SAXS). Our results show that the binding of ppGpp to BipA does not induce a nucleotide-specific conformational change, suggesting that both the ppGpp nucleotide and the small ribosomal subunit must be present to switch the binding specificity of BipA.

## Experimental Procedures

### 

#### 

##### Cloning, Protein Expression, and Protein Purification of Full-length BipA

The DNA sequence of full-length *bip*A (residues 1–607) from *E. coli* K12 MG1655 was inserted into pET28a vector (Novagen) between BamHI and XhoI restriction sites by in-fusion cloning (Clontech). Plasmid DNA encoding full-length BipA was transformed into *E. coli* T7 Express cells (New England Biolabs). Cells were grown in the presence of 30 μg/ml kanamycin in Lenox broth, and protein overexpression was induced with 0.2 mm isopropyl-β-d-thiogalactopyranoside when cells reached mid-log phase. Cells were grown for an additional 20 h at 16 °C, before being harvested, flash-frozen in liquid nitrogen, and stored at −80 °C until further use.

Cells containing overexpressed full-length BipA were resuspended in lysis buffer (25 mm HEPES-NaOH, 50 mm glycine-NaOH, pH 8.0) and lysed by passing the cell suspension three times through an EmulsiFlex-C3 homogenizer at 15,000 psi. Clarified cell lysate was loaded onto a 5-ml HisTrap column (GE Healthcare), washed with 1.5 m NaCl, and eluted with 200 mm imidazole. The 200 mm imidazole eluate was buffer-exchanged into lysis buffer before loading onto a 20-ml DEAE column (GE Healthcare). BipA protein was eluted from the column with a linear gradient of 0–600 mm NaCl. Protein content of each fraction was analyzed by SDS-PAGE. Fractions containing full-length BipA were pooled, concentrated, buffer-exchanged into storage buffer (10 mm HEPES-NaOH, 20 mm glycine-NaOH, pH 8.0), and stored at −80 °C until further use.

##### Cloning, Protein Expression, and Protein Purification of C-terminal Fragment of BipA

DNA sequence of C-terminal fragment of *bip*A (residues 306–607) from *E. coli* K12 MG1655 was inserted into pET28a vector (Novagen) between BamHI and XhoI restriction sites by in-fusion cloning (Clontech). Plasmid DNA encoding the C-terminal fragment of BipA was transformed into E. cloni® BL21 (DE3) cells (Lucigen). Cells were grown in the presence of 30 μg/ml kanamycin in MDAG medium (25 mm Na_2_HPO_4_, 25 mm KH_2_PO_4_, 50 mm NH_4_Cl, 5 mm Na_2_SO_4_, 2 mm MgSO_4_, 0.5% (w/v) glucose, 0.02% (w/v) each of the 20 amino acids, Asp, 0.25% Asp, 10 μm FeCl_3_) (14), and protein overexpression was induced with 0.5 mm isopropyl-β-d-thiogalactopyranoside when cells reached mid-log phase. Cells were grown for an additional 20 h at 22 °C before being harvested, flash-frozen in liquid nitrogen, and stored at −80 °C until further use.

Cells containing the overexpressed C-terminal fragment of BipA were resuspended in a lysis buffer (20 mm Tris-HCl, 100 mm NaCl, 0.28 mm PMSF, pH 8.0) and lysed by passing the cell suspension five times through EmulsiFlex-C3 homogenizer at 7,500 psi. Clarified lysate was loaded onto a 5-ml HisTrap column (GE Healthcare) and successively washed with 15 column volumes of lysis buffer, 500 mm NaCl, and 20 mm imidazole. The BipA C-terminal fragment was eluted from the column with a linear gradient of 20–300 mm imidazole. Fractions containing the BipA fragment with more than 95% purity were pooled, concentrated, buffer-exchanged into storage buffer (5 mm Tris-HCl, pH 8.0), and stored at 4 °C until further use.

##### Crystallization

Full-length BipA was crystallized by vapor diffusion out of a sitting drop consisting of a 1:1 ratio of 6 mg/ml full-length BipA to well solution (100 mm Tris-HCl, 2% (w/v) PEG 6000, and 5 mm [Co(NH_3_)_6_]Cl_3_, pH 8.0) at 20 °C within 3 days. Crystal quality was enhanced by micro-seeding to yield crystals with dimensions of up to 800 × 100 × 100 μm. Complexes of BipA with either GDP or ppGpp (TriLink) were formed by soaking crystals of full-length BipA overnight in a solution of 100 μm GDP or 50 μm ppGpp in the presence of 1 mm magnesium acetate, respectively. Crystals were stabilized by the successive addition of well solution with increasing concentration of ethylene or propylene glycol. Once the final glycol concentration reached 45% (v/v), crystals were flash-frozen in liquid nitrogen.

Crystals of the BipA C-terminal fragment were grown by sitting drop vapor diffusion from a solution consisting of a 10:1 ratio of 7 mg/ml protein to well solution (3.04 m sodium formate, 200 mm Tris-HCl, and 1 mm magnesium bromide, pH 7.6). Within 3–7 days, the crystals reached full size of 1,000 × 550 × 300 μm. Crystals were soaked in well solution containing an additional 30% (v/v) propylene glycol for 10 s before flash-freezing in liquid nitrogen.

##### Data Collection and Structure Determination

X-ray crystal diffraction data of full-length BipA of apo and nucleotide-bound states were collected on beamlines 5.0.1 and 5.0.2 at the Advanced Light Source (ALS) at the Lawrence Berkeley National Laboratory (LBNL). Collected data were processed with HKL-2000 (15) and Mosflm (16). The apo structure was solved by molecular replacement using Phaser-MR (17) as implemented in PHENIX (18) and a search model consisting of the N-terminal half of *E. coli* LepA (Protein Data Bank (PDB ID: 3CB4) and the structure of the C-terminal fragment of *Vibrio parahaemolyticus* BipA (PDB ID: 3E3X). The structures of nucleotide-bound states were solved by molecular replacement using the apo full-length BipA structure as an initial search model.

X-ray crystal diffraction data of the BipA C-terminal fragment were collected on beamline 24-ID-C at the Advanced Photon Source (APS) at the Argonne National Laboratory (ANL). Data were processed with Mosflm (16). Structure was solved by molecular replacement using Phaser-MR (17) in PHENIX (18) and the structure of the BipA C-terminal fragment of *V. parahaemolyticus* (PDB ID: 3E3X) as a search model. After initial refinement of the molecular replacement solution, the phase quality was improved by density modification.

Model bias of each refined solution was minimized by calculating composite omit maps with simulated annealing of torsion angles as implemented in PHENIX (18). Each model was rebuilt with Coot (19) and refined with Refmac5 (20) and PHENIX (18) in iterative cycles until *R*-factor values converged. All molecular structure figures were prepared with PyMOL (Version 1.7.4, Schrödinger).

##### Isothermal Titration Calorimetry

The thermodynamic parameters of the binding of nucleotides (ppGpp and GDP) to BipA were determined by using a MicroCal iTC200 system (GE Healthcare). Experiments were performed in duplicate at different temperatures ranging from 10 to 30 °C in 10 mm HEPES-NaOH, 20 mm glycine-NaOH, 100 mm NaCl, 5 mm MgCl_2_, pH 8.0. Each protein (∼0.1 mm) was titrated with 1 mm nucleotides in 2.6-μl aliquots dispensed over 5.2 s under continuous stirring at 700 rpm. The isothermal titration curves were processed with ORIGIN 7.0 (GE Healthcare). The data were analyzed by applying a single binding-site assumption and a non-linear regression to the integrated data, enthalpy change (Δ*H*), and affinity constant (*K_a_*) for each protein nucleotide titration.

##### Small-angle X-ray Scattering Data Collection and Data Analysis

An incident wavelength of 1.0 Å was used, and scattering data were collected at the SIBYLS beamline 12.3.1 at the ALS, LBNL, on a MAR 165 CCD area detector, within a *q* range of 0.01–0.32 Å^−1^ (*q* = 4π sin(θ/2)/λ, where θ is the scattering angle and λ is the wavelength) (21, 22). Data sets were collected on 15-μl sample volumes placed in a 1-mm-thick cuvette, and data for each sample and for exact buffer blanks were collected over four exposures of 0.5, 1, 2, and 4 s. BipA full-length data were collected at 1, 2, and 3 mg/ml to determine the optimal concentration for further analyses and to confirm a lack of concentration effects. The optimal 2 mg/ml concentration was used to collect data of BipA in the presence or absence of 300 μm guanosine nucleotide in the same buffer condition as used for isothermal titration calorimetry (10 mm HEPES-NaOH, 20 mm glycine-NaOH, 100 mm NaCl, 5 mm MgCl_2_, pH 8.0). SAXS data were processed and analyzed with ScÅtter 2.0 software.

## Results

### 

#### 

##### Crystal Structure of Full-length BipA

**FIGURE 1.**
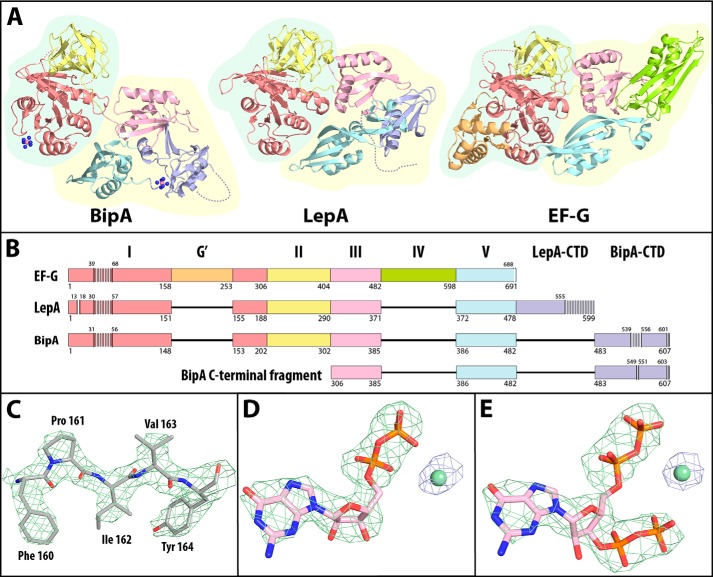
**Structure of full-length BipA in apo, GDP-, and ppGpp-bound forms.** *A*, structures of BipA, LepA (PDB ID: 3CB4), and EF-G (PDB ID: 1FNM) with common domains I, II, III, and V displayed in *red*, *yellow*, *pink*, and *blue*, respectively. The EF-G specific domains, G′ and IV, are in *orange* and *green*, respectively. The LepA and the BipA specific C-terminal domains are in *purple*. The cobalt cation ions and their coordinated amine molecules are displayed as *pink* and *blue spheres*, respectively. Disordered regions are indicated with *dashed lines. B*, sequence alignment of EF-G, LepA, BipA, and C-terminal fragment of BipA with domains color-coded as in *A*. Residue number below the sequence indicates residue N-terminal to the boundary between domains. The beginning and end of disordered regions are indicated with the residue number of the last ordered residue on the *top* of the sequence. Disordered regions are displayed as an *array of thin boxes. CTD*, C-terminal domain. *C*, a representative section of the 2D *F_o_* − *mF_c_* electron density map of apo full-length BipA contoured at 2.5 σ displayed in *green mesh. D* and *E*, unbiased *F_o_* − *F_o_* electron density map of the GDP-bound and ppGpp-bound BipA crystal structures, with GDP and ppGpp contoured at 4.0 σ in *green mesh* and the nucleotide-bound magnesium contoured at 6.0 σ in *blue mesh*.

Full-length protein of *E. coli* BipA crystallizes in the P2_1_ space group with two copies in the asymmetric unit, but is monomeric in solution under physiologically relevant salt concentrations as demonstrated by gel filtration (data not shown). The structure was solved and refined to a resolution of 2.6 Å as described under “Experimental Procedures”. (For a representative section of the final electron density map, see Fig. 1*C*. For further data collection and crystallography statistics, see Table 1.)

**TABLE 1:** **Data collection and refinement statistics**

	BipA, apo	BipA-GDP	BipA-ppGpp	BipA, CTF*^a^*
**Data collection**				
Beamline	5.0.2*^b^*	5.0.2*^b^*	5.0.1*^b^*	24-ID-C*^c^*
Wavelength (Å)	1.0000	1.0000	0.9800	0.9792
Resolution range (Å)	50.0–2.6 (2.68–2.63)*^d^*	89.2–3.1 (3.25–3.06)	79.8–3.3 (3.58–3.31)	48.0–2.5 (2.58–2.48)
Space group	P2_1_	P2_1_	P2_1_	P4_1_2_1_2
Cell dimensions				
a, b, c (Å)	56.7, 161.4, 89.6	56.4, 160.0, 90.0	56.6, 159.6, 89.6	83.6, 83.6, 191.8
α, β, γ (°)	90.0, 98.0, 90.0	90.0, 97.7, 90.0	90.0, 98.0, 90.0	90.0, 90.0, 90.0
Total reflections	46,793 (2,277)	111,672 (18,078)	69,563 (13,671)	186,562 (14,549)
Unique reflections	12,647 (785)	29,871 (4,772)	23,189 (4,717)	24,915 (2.697)
Completeness (%)	99.7 (96.1)	99.3 (98.8)	98.9 (98.7)	99.8 (98.7)
Mean *I*/σ	12.4 (1.4)	8.6 (2.0)	7.0 (1.8)	6.5 (1.0)
Multiplicity	3.7 (2.9)	3.7 (3.8)	3.0 (2.9)	7.5 (5.4)
*R*_merge_ (%)	10.6 (91.9)	10.3 (67.6)	17.3 (70.1)	14.5 (130.8)

**Refinement**				
Resolution range (Å)	46.00–2.63	48.18–3.06	48.47–3.31	47.96–2.48
*R*_work_/*R*_free_ (%)	21.0/24.8	23.2/27.1	23.3/29.1	19.4/22.7
r.m.s.d. bond length (Å)	0.003	0.003	0.005	0.010
r.m.s.d. bond angles (°)	0.744	0.741	1.023	1.286
Ramachandran plot				
Most favored (%)	93.5	92.2	91.3	95.9
Additional allowed (%)	5.9	7.4	7.0	4.1
Disallowed (%)	0.6	0.4	1.7	0.0
Wilson B factor (Å^2^)	58.9	72.7	70.4	70.4
Average B factor (Å^2^)	78.0	96.0	104.0	86.0
**PDB entry**	4ZCI	4ZCL	4ZCM	4ZCK

*^a^* CTF is the C-terminal fragment of BipA consisting of domains III and V and the BipA specific C-terminal domain.

*^b^* Beamline 5.0.1 and 5.0.2 are run by the Berkeley Center for Structural Biology at the Advanced Light Source.

*^c^* Beamline 24-ID-C is run by the Northeastern Collaborative Access Team at the Advanced Photon Source.

*^d^* Values in parentheses are for the highest resolution shell.

Full-length BipA can be subdivided into five domains. Beginning from the N terminus, the first four domains are homologous to domains I, II, III, and V of EF-G, whereas the last domain at the C terminus is distinct and protein-specific (Fig. 1, *A* and B). The domains of BipA, like those of EF-G and homolog LepA, appear to group together into two superdomains, the first of which is formed by domains I and II, and the second of which is formed by domains III, V, and the BipA-specific C-terminal domain (23, 24). Domain I (residues 1–202) consists of a central six-stranded β-sheet surrounded by five α-helices, which is characteristic for the G domain found in translational GTPases (25). Domain II (residues 203–302) has the distinctive OB-fold of oligonucleotide/oligosaccharide binding domains. Domains III (residues 303–385) and V (residues 386–482) both have the same double-split β-α-β fold (26). The unique C-terminal domain of BipA (residues 483–607) spatially occupies a position between domains III and V and consists of a unique mixed α + β fold composed of eight β-sheets and two α-helices. A long, polar region (residues 540–555) that extends from the distal end of the C-terminal domain is disordered in the crystal structure. A similarly localized disordered region in the structure of apo LepA adopts a functionally significant helix-turn-helix structure when bound to the ribosome (27). We obtained crystals of full-length BipA only in the presence of [Co(NH_3_)_6_]^3+^, two of which can be localized within the structure. One cation is in the center of a crystal contact at the surface of domain I, whereas the other lies in the loop that connects domain V and the C-terminal domain (Fig. 1*A*). Despite multiple attempts, we were unsuccessful in replacing the cobalt ions with magnesium ions.

##### Crystal Structure of the Isolated BipA C-terminal Fragment

**FIGURE 2.**
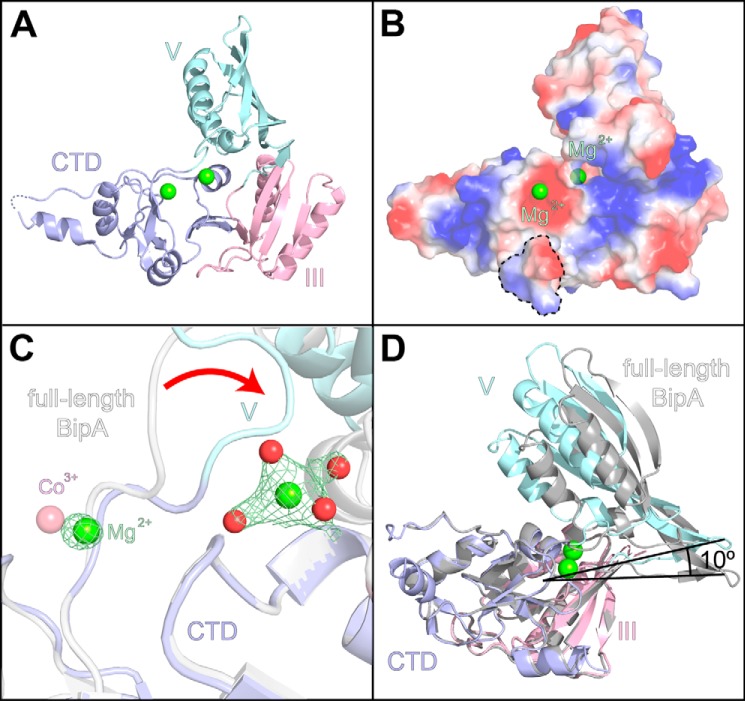
**Structure of C-terminal fragment of BipA consisting of domain III, V, and C-terminal domain.** *A*, overall view of the C-terminal fragment with protein rendered as a schematic and ligated magnesium ions as green spheres. *CTD*, C-terminal domain. *B*, surface charge distribution of the C-terminal domain within the C-terminal fragment with positive charges displayed in *blue* and negative charges displayed in red. The C-terminal helix essential for ribosome binding is outlined with a *dashed line. C*, 2D *F_o_* − *mF_c_* electron density map of the two proximal magnesium ions (*green spheres* with coordinated water molecules as *red spheres*) bound to the C-terminal domain of C-terminal fragment (*blue*) contoured at 1.0 σ in *green mesh*. The *pink sphere* indicates the position of cobalt ion bound to the C-terminal domain of the full-length BipA (*light gray*). *D*, superposition of the C-terminal fragment by itself and that derived from the full-length BipA structure. The domains of the C-terminal fragment structure are displayed in the same colors as in Fig. 1, except that all domains of the derived C-terminal fragment are displayed in *light gray*.

To evaluate the significance of the bound cations and to resolve the long, disordered, polar region, we set out to crystallize a C-terminal fragment of BipA consisting of domains III and V and the C-terminal domain in the presence of magnesium ions (Fig. 2*A*). This fragment of BipA crystallized in the P4_1_2_1_2 space group in the presence of 1 mm magnesium ions and diffracted to 2.5 Å. Based on the calculation of the Matthews coefficient, two molecules were expected in the asymmetric unit, but the native Patterson map and the self-rotation function did not reveal any non-crystallographic symmetry. Furthermore, molecular replacement with Molrep (28) and Phaser-MR (17) with different truncations of *V. parahaemolyticus* BipA (PDB ID: 3E3X) as search models found only reasonable solutions for one protomer in the asymmetric unit cell. The final structure was solved and refined as described under “Experimental Procedures” and converged to *R*_work_ of 19.4% and *R*_free_ of 22.7%.

The structure of the C-terminal fragment is similar to that of the corresponding part of the full-length protein structure and to the C-terminal fragment of *V. parahaemolyticus* BipA. However, domain V is rotated toward the interface of the C-terminal domain and domain III (Fig. 2*D*). In addition, nearly all of the long, basic, disordered region is now structured, and the position of the [Co(NH_3_)_6_]^3+^ ion in the full-length protein structure has been replaced by two proximal magnesium ions (Fig. 2*A*). One magnesium ion has supplanted the [Co(NH_3_)_6_]^3+^ ion, and the other is close to the pivot point of the rotation of domain V (Fig. 2*C*). This placement of the magnesium ions in the C-terminal domain increases the positive surface charge in the proximity of the conserved basic C-terminal helix essential for ribosome binding of BipA (29)(Fig. 2*B*). This also explains the need for the extensive washing of the immobilized C-terminal fragment during protein purification to release all the bound nucleic acid from the protein.

##### Crystal Structures of GDP- and ppGpp-bound BipA

To gain a better understanding of the effect of ppGpp on BipA, we solved the structure of full-length BipA in the presence of GDP to a resolution of 3.1 Å and in the presence of ppGpp to a resolution of 3.3 Å. In each of these structures, the nucleotide and the coordinated magnesium ion can be localized unambiguously in the initial unbiased difference electron maps (Fig. 1, *D* and E).

**FIGURE 3.**
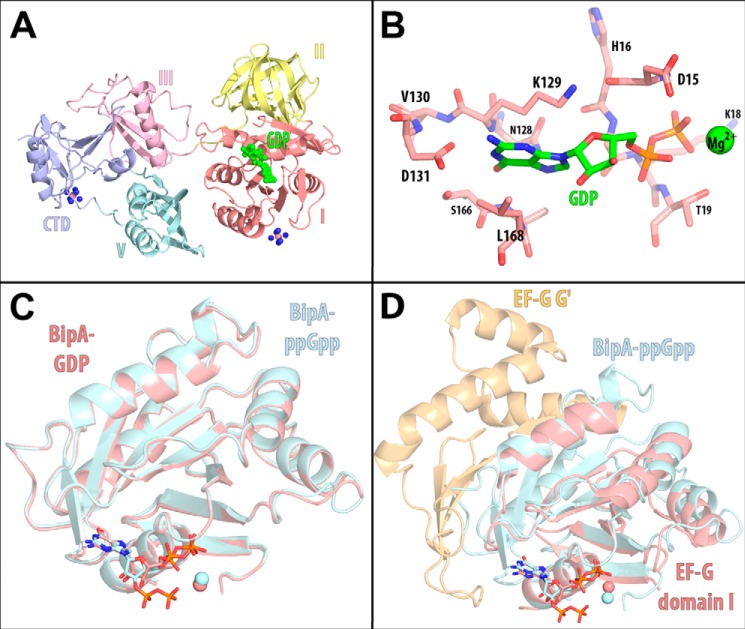
**Binding of GDP and ppGpp to full-length BipA.** *A*, overview of the GDP-bound BipA structure. The protein is rendered as a schematic with the domains colored as in Fig. 1. The bound GDP molecule is rendered as a space-filling model and colored *green. B*, close-up view of the GDP binding pocket in domain I. Residues of protein ^128^NKVD^131^, ^166^SAL^168^, and ^15^DHGKT^19^ form the binding pocket for GDP and are displayed in *red*. The bound GDP is in *green*, and the bound magnesium ion is represented as a *green sphere. C*, superposition of domain I of the GDP-bound and of the ppGpp-bound structures. The GDP-bound form is displayed in *red*, and the ppGpp-bound form is in *blue. D*, superposition of domain I of GDP-bound EF-G and ppGpp-bound BipA structures. EF-G domains I and G′ are displayed in *red* and *orange*, and the BipA domain I is in *blue*.

The structure of BipA with bound GDP is very similar to that of the apo form with a root mean square deviation (r.m.s.d.) of 0.49 Å across all matching main chain atoms. The GDP nucleotide is bound in domain I (Fig. 3*A*) in similar way as in other translational GTPases. The binding pocket for the nucleotide's base is composed of the conserved sequence motifs NKVD (residues 128–131) and SAL (residues 166–168). These residues provide hydrogen bonds and hydrophobic interactions for the selective binding of the base. The conserved residues of the G1-box sequence motif (residues ^12^AHVDHGKT^19^) wrap around the phosphate groups of GDP with the side chain of Asp^15^ coordinating a magnesium ion that in turn coordinates to both phosphates of GDP (Fig. 3*B*). The “switch 1” region of BipA (residues 42–65), whose position is affected by the γ-phosphate of GTP in other GTPases, is disordered (30).

Surprisingly, the structure of BipA with bound ppGpp is very similar to that of BipA with bound GDP, reflected in a low r.m.s.d. of 0.28 Å across all matching main chain atoms. Even the GDP portion of ppGpp is in the same structural environment as GDP by itself, forming the same interactions. The additional 3′-pyrophosphate of ppGpp extends toward the 5′-pyrophosphate group chelating a magnesium ion between them (Fig. 3*C*). However, to exclude possible constrictions of conformational freedom of BipA due to crystal formation, we investigated the conformation of BipA in solution by ITC and by SAXS.

##### Isothermal Titration Calorimetry of BipA with GDP and ppGpp

**FIGURE 4.**
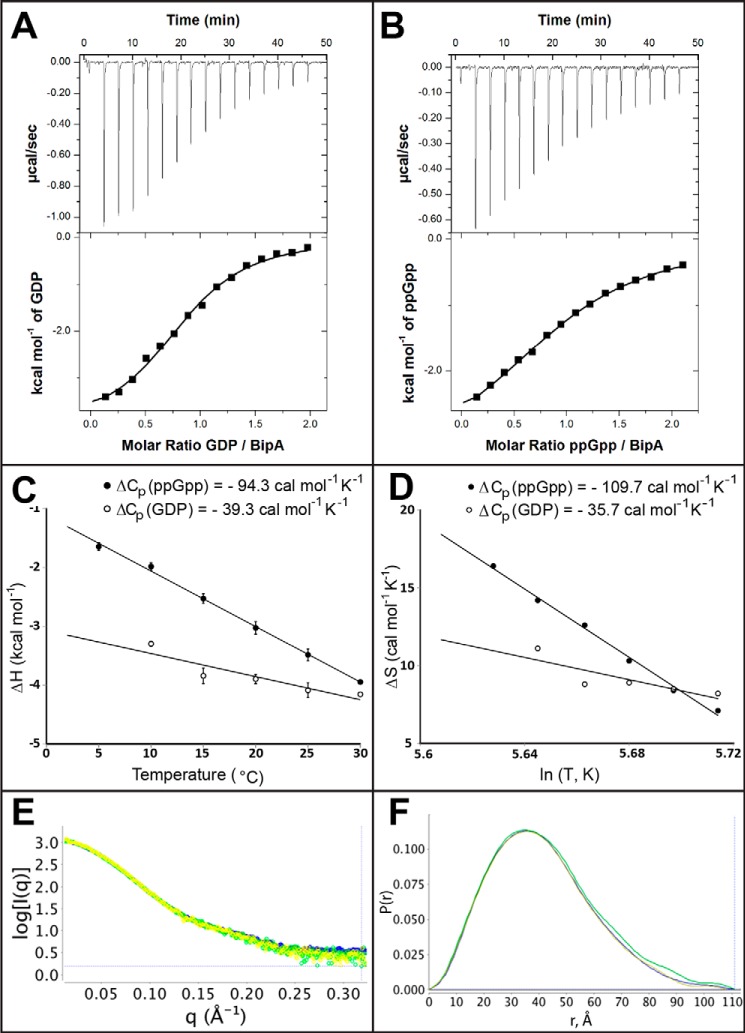
**ITC and SAXS analysis of nucleotide binding to BipA.** *A* and *B*, isothermal titration calorimetry curves of BipA titrated with GDP (*A*) and ppGpp (*B*) after correction of the dilution effect of nucleotides. *C*, enthalpy change for the GDP (*open circles*) and ppGpp binding (*filled circles*) to BipA as a function of temperature (°C). *D*, entropy change for the GDP (*open circles*) and ppGpp binding (*filled circles*) to BipA as a function of the logarithm of temperature in Kelvin. The heat capacity change (Δ*C_p_*) value from the enthalpy (*C*) is similar to that obtained from entropy (*D*). *E*, the unique scattering profiles of the apo BipA, in *yellow*, BipA with bound GDP, in *green*, and BipA with bound ppGpp, in *blue*, are shown with intensity (*I*), plotted against the photon momentum transfer (*q*). *F*, the P(r) distribution functions of apo BipA, in *yellow*, BipA with bound GDP, in *green*, and BipA with bound ppGpp, in *blue*, are depicted.

The switch of the binding preference of BipA from ribosomes to the small ribosomal subunits during starvation suggests a possible conformational change of BipA upon binding of ppGpp (11). We used ITC to estimate binding affinities, enthalpies, and entropies for the binding of ppGpp and of GDP to *E. coli* BipA at temperature intervals of 5–30 and 10–30 °C, respectively (Table 2). A typical titration curve for GDP and for ppGpp is shown in Fig. 4, *A* and *B*, respectively. The binding affinity of BipA for GDP is only slightly stronger than that for ppGpp. Plotting the measured binding enthalpy values against temperature yields the heat capacity change (Δ*C_p_*) due to nucleotide binding from the slope. The Δ*C_p_* estimates for GDP and ppGpp binding to BipA are −39.3 and −94.3 cal mol^−1^ K^−1^, respectively (Fig. 4*C*). Similar values of Δ*C_p_* for GDP and ppGpp binding were obtained using the temperature dependence of the binding entropy (Fig. 4*D*). On the assumption of additive contributions of partial heat capacities (31), a comparison of Δ*C_p_* upon binding of GDP and of ppGpp allows us to ascertain the contribution of the 3′-pyrophosphate group to Δ*C_p_* for ppGpp binding with −55.0 cal mol^−1^ K^−1^.

**TABLE 2:** **Thermodynamic parameters for ppGpp and GDP binding to BipA and EF-G. All samples were determined in 10 mm HEPES-NaOH, 20 mm Glycine-NaOH, 5 mm MgCl_2_, 100 mm NaCl, pH 8.0. Data for IF2 and EF-G were taken from Refs. 31, 32, and 39**

Protein	Ligand	Temperature	*K_a_*	Δ*H*	*T*Δ*S**^a^*	Δ*G**^b^*
		°*C*	*m*^−*1*^	*kcal/mol*	*kcal/mol*	*kcal/mol*
BipA	GDP	10	9.5 ± 0.6 × 10^4^	−3.30 ± 0.05	3.14	−6.44
		15	6.7 ± 0.8 × 10^4^	−3.84 ± 0.13	2.53	−6.37
		20	7.0 ± 0.6 × 10^4^	−3.90 ± 0.08	2.61	−6.51
		25	6.9 ± 0.7 × 10^4^	−4.09 ± 0.13	2.52	−6.60
		30	6.0 ± 0.4 × 10^4^	−4.16 ± 0.06	2.48	−6.63
	ppGpp	5	7.6 ± 1.0 × 10^4^	−1.65 ± 0.07	4.56	−6.20
		10	4.3 ± 0.4 × 10^4^	−1.98 ± 0.06	4.02	−6.00
		15	4.7 ± 0.4 × 10^4^	−2.53 ± 0.08	3.63	−6.16
		20	3.2 ± 0.3 × 10^4^	−3.02 ± 0.11	3.02	−6.04
		25	2.4 ± 0.2 × 10^4^	−3.48 ± 0.10	2.50	−5.99
		30	2.5 ± 0.3 × 10^4^	−3.95 ± 0.19	2.15	−6.09
IF2	GDP*^c^*	25	6.1 × 10^5^	−12.50	−4.62	−7.88
	ppGpp*^d^*	25	3.6 × 10^5^	−12.81	−5.24	−7.57
EF-G	GDP*^e^*	25	1.1 × 10^5^	−5.90	0.96	−6.86
	ppGpp*^d^*	25	7.2 × 10^4^	−5.09	1.53	−6.62

*^a^* Δ*G* (the change of Gibbs free energy) value was obtained from equation: Δ*G* = -*RT*ln*K_a_*.

*^b^ T*Δ*S* (the change of entropy) value was obtained from equation: Δ*G* = Δ*H* − *T*Δ*S*.

*^c^* Data from Ref. 39.

*^d^* Data from Ref. 31.

*^e^* Data from Ref. 32.

By way of comparison, the previously published Δ*C_p_* estimates for the binding of GDP or GTP to EF-G are −21 or −270 cal mol^−1^ K^−1^, respectively (32). The calculated γ-phosphate contribution to the heat capacity change of −249 cal mol^−1^ K^−1^ was interpreted as the solvent protection of about 15 residues, probably distributed over two loops of the GTPase domain (*i.e.* switch 1 and 2). The small numerical value of Δ*C_p_* for GDP binding was interpreted as reflecting the absence of a conformational change of EF-G upon GDP binding (32). We, therefore, similarly conclude that the presence of the 3′-pyrophosphate of ppGpp does not induce a conformation in BipA different from that seen in the presence of GDP, a conclusion consistent with the above x-ray crystal structures. However, to exclude compensatory effects masking a larger conformational change, we analyzed the change of the conformation of BipA due the presence of ppGpp and GDP in solution by SAXS.

##### Small-angle X-ray Scattering of BipA in GDP- and in ppGpp-bound Forms

To determine the effects GDP or ppGpp binding on the BipA conformation in solution, we conducted SAXS analyses of the full-length BipA protein. Measurements were performed with 2 mg/ml proteins at 20 °C, both in the absence and in the presence of 300 μm GDP or ppGpp. At these nucleotide concentrations, more than 90% of the BipA molecules have bound nucleotide (Fig. 4*E*). All the SAXS samples were monodisperse, as confirmed by gel filtration and by the observed linearity of the Guinier plots. The radius of gyration (*R_g_*), which characterizes the particle size of a sample, was derived from the SAXS data using both the Guinier plot and the electron pair-distance distribution function, P(r). These analyses of the data revealed little conformational difference between the samples. Apo BipA has a Guinier *R_g_* of 31.06 Å and a real-space *R_g_* of 33.18 Å with a maximum dimension (*D*_max_) of 108 Å. GDP-bound BipA has similar values of 32.02 and 32.68 Å for Guinier and real-space *R_g_* values, respectively, and a *D*_max_ of 107 Å, as does ppGpp-bound BipA with a Guinier *R_g_* of 32.21 Å and real-space *R_g_* of 32.94 Å and a *D*_max_ of 111 Å. The electron pair-distance distribution function, P(r), reflects the distribution of all distances between two electrons within a molecule. The superimposable electron pair-distance distribution functions P(r) for apo BipA and BipA in the presence of GDP or ppGpp (Fig. 4*F*) also suggest similar solution conformations.

## Discussion

The switch of the binding preference of BipA from ribosomes to small ribosomal subunits in the presence of elevated concentration of ppGpp suggests that ppGpp could allosterically regulate the binding specificity of BipA. Allosteric regulation is usually accomplished by a conformational change of a protein upon binding of an effector molecule. Therefore, we determined the structure of the apo, GDP-, and ppGpp-bound forms of BipA to ascertain whether ppGpp binding controls the affinity of BipA for the small subunit via a conformational change.

The x-ray crystal structure of the apo form of BipA consists of two superdomains. The N-terminal superdomain comprises domains I and II, and the C-terminal superdomain comprises domains III and V and the unique C-terminal domain. In BipA, these superdomains adopt a distinctive relative orientation to each other that has not been observed in any structure of the homologs EF-G or LepA (Fig. 1*A*). The isolated C-terminal fragment of BipA adopts an even more compact unit, occupying a smaller volume in the presence of magnesium (Fig. 2*D*). This observed compaction suggests that the C-terminal superdomain forms a functional unit even on the ribosome, similar to the superdomain structure seen in the crystal structure of EF-G bound to ribosomes (24). Such a functional unit could also explain the loss of ribosome-stimulated GTPase activity of C-terminal truncated BipA (29). Modeling the structure of the isolated C-terminal fragment into the structure of the full-length protein results only in minor clashes between domains I and V. The presence of additional magnesium ions in the C-terminal domain increases the positive charge near the regions of the protein predicted to interact with ribosomes (29) (Fig. 2*B*).

Surprisingly, the structures of GDP- and ppGpp-bound BipA are fundamentally similar to that of the nucleotide-free protein (Fig. 3*A*). Similarly, the solution structure of BipA with bound GDP or ppGpp yields the same electron pair distance distribution (Fig. 4*F*). NMR studies of the GTPase domain of IF2 confirm that the GTPase domain adopts the GDP-bound conformation upon ppGpp binding in solution (33). In the case of BipA, it is not simply the GTPase domain by itself, but the whole protein that adopts the same conformation upon binding of GDP or ppGpp. Our thermodynamic measurements also suggest that the presence of 3′-pyrophosphate on ppGpp is insufficient to overcome the GDP conformation.

Superposing the structure of the GTPase domain of BipA with bound ppGpp on that of EF-G with bound GDP (PDB ID: 1FNM) reveals that EF-G can accommodate ppGpp without any further structural adjustments (Fig. 3*D*). These structural similarities are reflected in the similar binding affinity of BipA and of EF-G for GDP and ppGpp (Table 2). Although the binding affinities are similar, the Gibbs energy change (Δ*G*) is partitioned differently between enthalpy change (Δ*H*) and entropy change (Δ*S*) for the binding of ppGpp to BipA and to EF-G, hinting at possibly different contributions that determine the binding affinities of guanosine nucleotides to these two proteins. Our model of EF-G with bound ppGpp concurs with the observation that the apo, GDP-, and GTP analog-bound forms have the same structure in crystal (34–36) and in solution (37).

Our results indicate that the interactions with both ppGpp and the small ribosomal subunit are necessary in order for BipA to alter its binding preferences. A similar mechanism was proposed for the binding of GTP to other translational GTPases (38). This suggests a common pattern for the regulation of the binding affinity of translational GTPases, in which the change of the binding preference of the GTPase is only accomplished in the presence of both the nucleotide and the corresponding binding partner, *i.e.* aminoacyl-tRNA for EF-Tu, ribosome for EF-G, or small ribosomal subunit for BipA.

As BipA is only critical for biogenesis of large ribosomal subunits at low temperature, we believe that a drug that stabilizes the GDP conformation of BipA will not affect the biogenesis of large ribosomal subunits during an infection. However, a stabilized GDP conformation would prevent the binding of BipA to the small subunit in the presence of ppGpp, therefore providing a novel avenue for drug discovery efforts that aim to inhibit virulence specifically to reduce the selective pressure for resistance mutations.

## Author Contributions

J. H. and S. D. developed purification and crystallization protocol of full-length and C-terminal fragment of BipA, respectively. H. F. collected and solved the x-ray crystal structure, performed ITC measurements, prepared samples for SAXS, made all the figures, and wrote the initial draft of the manuscript. J. J. P. P. designed SAXS experiments and performed data analysis. G. B. conceived and helped design all experiments. H. F., J. J. P. P., and G. B. analyzed all data and results and wrote the final version of paper. All authors approved the final version of the manuscript.

## Footnotes

2 The abbreviations used are:

EF: elongation factor
ppGpp: guanosine-3′, 5′-bis pyrophosphate
ITC: isothermal titration calorimetry
SAXS: small-angle x-ray scattering
r.m.s.d.: root mean square deviation.
